# Transdermal Delivery of Botulinum Neurotoxin A: A Novel Formulation with Therapeutic Potential

**DOI:** 10.3390/pharmaceutics17020146

**Published:** 2025-01-22

**Authors:** Raj Kumar, Bal Ram Singh

**Affiliations:** Institute of Advanced Sciences, Dartmouth, MA 02747, USA; bsingh@inads.org

**Keywords:** topical, botulinum neurotoxin, stratum corneum, epidermis, drug delivery

## Abstract

Background: Botulinum neurotoxin is widely regarded as a “wonder medicine” due to its therapeutic efficacy in treating a variety of conditions. While it is traditionally classified as a neurotoxin, it is arguably more appropriate to refer to it as a neuromedicine. All FDA-approved formulations of botulinum neurotoxin are currently administered through intramuscular injections, with no other delivery methods widely used. The primary reasons for this include the following: (a) the extremely high potency of the toxin, (b) the potential for diffusion to adjacent muscles, (c) factors related to the site of administration (e.g., muscle thickness), (d) the large size of the molecule, (e) the impermeability of skin to large protein molecules, and (f) safety concerns. Despite these challenges, there is growing interest in the development of an effective transdermal formulation of botulinum neurotoxin. Refining and standardizing the delivery technology for topical or transdermal use remains an important goal for the future. Methods: The aim of this study was to develop a nanoemulsion-based transdermal formulation capable of delivering active botulinum neurotoxin (BoNT) through human skin. The goal was to demonstrate its efficacy in a mouse model, highlighting the therapeutic effects on both neuromuscular activity and hyperhidrosis. We successfully developed a nanoemulsion-based formulation that facilitates the transdermal delivery of BoNT. The formulation was homogeneous, stable, and efficacious. In a mouse model, we evaluated the neurotoxin’s impact on neuromuscular function using the Digital Abduction Score (DAS) for toe-spread and rota-rod assay to assess motor coordination. Results: The results confirmed the successful paralytic effect of the neuotoxin. The formulation significantly reduced sweating in the hyperhidrosis mouse model, indicating the therapeutic potential for this indication. Beyond the neurotoxin’s paralyzing effect, we also observed the recovery of nerve function, showing that the neurotoxin does not cause permanent damage, further underscoring its safety and efficacy. Conclusions: This formulation is the first of its kind to successfully deliver a large biomolecule like BoNT across the skin and produce a therapeutic effect. The ability to deliver large biomolecules transdermally has the potential to serve as a platform technology for treating a variety of conditions, including neuromuscular disorders, skin conditions, and localized pain management.

## 1. Introduction

Skin, the body’s largest organ, serves as the primary line of defense and is the most accessible organ due to its extensive surface area. It protects the body from microbial invasion, external pathogens, and environmental hazards. In addition to its protective role, the skin is involved in various physiological functions, including the regulation of body temperature; storage of water and lipids; prevention of water loss; sensory and motor responses through the detection of touch, heat, and cold; and the synthesis of vitamin D. Human skin is composed of approximately 70% water, 27.5% proteins, 2% lipids, 0.5% mineral salts, and trace elements. The skin is structured into three main layers: (a) the epidermis (the outermost layer); (b) the dermis (beneath the epidermis); and (c) the hypodermis (the deeper subcutaneous layer). The epidermis, the thinnest and outermost layer, is primarily composed of squamous cells in the stratum corneum, basal cells in the second layer, and melanocytes located at the base of the epidermis. The dermis, the middle layer, contains blood and lymphatic vessels, hair follicles, sweat glands, sebaceous glands, collagen fibers, fibroblasts, nerves, and sensory receptors for pain and touch. This layer provides strength and elasticity to the skin and is primarily maintained by structural protein collagen. The hypodermis, the deepest layer, is composed mainly of collagen and adipose tissue and functions as a shock absorber.

The skin, due to its accessibility, has been explored as a potential route for the delivery of therapeutic molecules into systemic circulation. However, the skin’s formidable barrier properties, particularly against large molecules like proteins, pose significant challenges for transdermal delivery. Despite these challenges, various strategies have been developed to enhance the permeability of the skin to small molecules and peptides. Techniques such as chemical enhancers, iontophoresis, microneedles, electroporation, sonophoresis, thermal and laser ablation, radiofrequency ablation, and non-invasive jet injectors have been employed to facilitate molecular penetration [1,2,3,4,5,6,7]. While transdermal delivery offers a promising noninvasive alternative, it remains limited for the delivery of large macromolecules, such as proteins. In the present study, we demonstrate the successful transdermal delivery of Botulinum neurotoxin A (BoNT/A, 150 kDa) without the use of any enhancement techniques.

### 1.1. Botulinum Neurotoxin

There are seven major serotypes of Botulinum neurotoxins (BoNTs), each consisting of two principal domains: a binding and translocation domain (Heavy Chain [HC]) and a catalytic domain (Light Chain [LC]), which are linked by a disulfide bond [8]. Upon binding to the presynaptic nerve membrane, BoNT is internalized via endocytosis, and the LC is translocated across the membrane through a pore formed by the translocation domain (TD) of the HC [9]. The LC of each BoNT serotype is a zinc-metalloprotease that functions as an endopeptidase with high substrate specificity, requiring a relatively long peptide sequence, which varies depending on the serotype [10]. This specificity is a unique feature of BoNTs, as other microbial metalloproteases can recognize shorter sequences, such as dipeptides [10,11]. Serotype A and E cleave SNAP-25 (25-kDa synaptosomal-associated protein), while serotypes B, C, D, F, and G cleave synaptobrevin. Additionally, serotype C cleaves both SNAP-25 and syntaxin, all of which are involved in the process of exocytosis [12].

The Botulinum neurotoxin A complex is the most potent known toxin to humans, with an LD50 in mice (1 unit) ranging from 25 to 50 picograms. In molar terms, Botulinum neurotoxin A complex is approximately 1.8 billion times more lethal than diphtheria toxin, 600 million times more lethal than sodium cyanide, 30 million times more lethal than cobra venom, and 12 million times more lethal than cholera toxin. Due to its extraordinary potency, BoNT-A is widely used as a therapeutic agent for treating various physiological conditions [12,13]. In clinical practice, BoNT-A is available both in its complex form (Botox^®^, Dysport^®^, and Myobloc^®^) and in its pure dichain form (Xeomin^®^).

### 1.2. Administration of Botulinum Neurotoxin

Botulinum neurotoxin (BoNT) is increasingly recognized as a therapeutic biomolecule and has been approved by the FDA for several medical indications. These include chronic migraine, cervical dystonia, blepharospasm, strabismus, hyperhidrosis, cosmetic applications, urinary incontinence due to detrusor overactivity, and hemifacial spasms. Beyond these approved indications, BoNT is commonly used off-label for over 800 neuro-muscular conditions [14].

Botulinum neurotoxin is typically administered via injection of a BoNT-containing formulations into the affected muscles or glands. The effective dose generally depends on the size of the target muscle or gland, with larger muscles often requiring higher doses; however, the susceptibility to BoNT may vary among individuals [15]. The local injection of BoNT works by weakening overactive muscles and reducing hypersecretion from glands that are innervated by cholinergic neurons. These local injections have proven effective for a variety of conditions, including achalasia, chronic anal fissures, acne scars, and hyperhidrosis.

Despite its therapeutic efficacy, injection-based administration can be associated with side effects such as pain, erythema, bruising, tenderness, and risks linked to needle penetration. These drawbacks have prompted interest in exploring alternative modes of delivery, such as topical or transdermal administration, to improve patient comfort and expand therapeutic options.

### 1.3. Topical or Transdermal BoNT

The topical administration of Botulinum neurotoxin A (BoNT/A) allows for deeper penetration of the active toxin, potentially achieving effects similar to those of injectable formulations by inhibiting acetylcholine release and blocking neuromuscular transmission. While much of the focus on topical or transdermal delivery has been on small molecules, delivering large molecules such as proteins through the skin presents significant challenges. Several techniques have been developed to overcome the skin’s formidable barrier, particularly the stratum corneum. The delivery of large molecules often requires the use of adjunctive methods, such as iontophoresis, ultrasound, microneedles, or the incorporation of cell-penetrating peptides as carriers or conjugates. Currently, no formulation exists that can deliver BoNT without the aid of such enhancement techniques. Therefore, there remains a need for a formulation that contains a therapeutically effective amount of BoNT, capable of permeating the skin, delivering the toxin in appropriate quantities, and demonstrating clinical efficacy in producing the desired therapeutic effect.

In this study, we demonstrated the following: (a) the development of a homogeneous and stable BoNT formulation; (b) the successful transdermal delivery of BoNT/A (150 kDa) without the use of any enhancement techniques; (c) the confirmation of the efficacy of transdermal delivery through the Digital Abduction Score (DAS) of the mouse toe-spread assay; (d) the recovery of paralyzed toes as measured by the rota-rod assay; (e) the distribution of the neurotoxin through human skin; and (f) the validation of the transdermal delivery of BoNT/A in a therapeutic model.

## 2. Materials and Methods

### 2.1. Animals

All animal procedures were approved by the Institutional Animal Care and Use Committee (IACUC) of the facility, and animal handling during experiments followed the approved protocols. BALB/c mice (4–6 weeks old, average weight 22 ± 2 g, unless otherwise stated) were used in this study. Mice were group-housed (5 per cage) under a 12:12-h light–dark cycle in static polycarbonate micro-isolation rodent cages with paper chip bedding. Water and rodent chow were provided ad libitum, with supplemental food enrichment. Room temperature was maintained at 64–73 °F and relative humidity at 35–75%.

### 2.2. Purification of BoNT/A Toxin

Botulinum neurotoxin A (BoNT/A) was purified following the procedure described by DasGupta and Sathyamoorthy [16]. Briefly, the neurotoxin was produced in five 1-L glass bottles containing the following media: N-Z Amine Type A (2.0%), yeast extract (0.5%), and autolyzed yeast paste (0.6%), with the pH adjusted to 7.2 using 4 N NaOH. The medium was autoclaved (1 h at 121 °C), and 20% w/v glucose (autoclaved) was added to achieve a final concentration of 0.5%. Each bottle was inoculated with 100 µL of cultured fluid from a cooked meat medium and incubated without agitation at 37 °C for 96 h. After incubation, the pH was adjusted to 3.5 using 3 N H_2_SO_4_, and the precipitate was collected by centrifugation (6000 rpm, 10 min, 4 °C; Sorvall). The precipitate was extracted with 0.1 M citrate buffer (pH 5.5) for 1 h, centrifuged again (12,000 rpm, 10 min, 4 °C), and the supernatant collected. The pellet was re-extracted with citrate buffer, and both supernatants were pooled and precipitated with ammonium sulfate overnight. The resulting precipitate was collected by centrifugation (12,000 rpm, 20 min, 4 °C) and dissolved in 50 mM phosphate buffer (pH 6.0). This was followed by digestion with ribonuclease (50 µg/mL, 3 h at 37 °C). Column chromatography was then used to purify both the complex and pure neurotoxin [16].

The LD_50_ of the purified neurotoxin was determined by the intraperitoneal injection of serial dilutions of the lyophilized BoNT/A (reconstituted in 0.5 mL of sterile 0.2% type A gelatin in 30 mM sodium phosphate buffer, pH 6.2) into mice. The following concentrations were prepared: 10, 12.5, 15.63, 19.53, 24.42, 30.53, 38.15, 47.70, 59.63, 74.53, and 93.18 pg/mL. Five mice were used for each dilution, and 500 µL of neurotoxin solution was administered intraperitoneally. Mice were monitored for mortality at 72 and 96 h post-injection. LD_50_ was calculated using the method described by [17].

### 2.3. Preparation of Nano-Emulsion

Nano-emulsions, which are colloidal dispersions of two immiscible phases (oil and water), were prepared using the following components: propylene glycol (Specialized Rx, Blaine, MN, USA), phenoxyethanol (Millipore, Burlington, MA, USA), sodium hyaluronate (Thermo Fisher Scientific, Waltham, MA, USA), Tween-80 (Sigma Aldrich, Burlington, MA, USA), hydrogenated castor oil (Sigma Aldrich, Burlington, MA, USA), saponin (Sigma Aldrich, Burlington, MA, USA), and tricaprylin (TCI Chemical, Portland, OR, USA). The emulsifier saponin was first dissolved in water, followed by the addition of sodium hyaluronate, propylene glycol, and Tween-80, which were dissolved in water. Tricaprylin and phenoxyethanol were added last. Each component was mixed for 15 min to ensure proper dispersion and dissolution before the addition of the next. The emulsion was stored at 4 °C until use.

### 2.4. Formulation of Botulinum Toxin A

Botulinum neurotoxin A (0.3 mg) was mixed with the prepared nano-emulsion and stirred for 20–25 min at room temperature to form the stock solution. All subsequent dilutions were prepared by mixing the stock solution with the nano-emulsion. These final formulations are referred to as formulated neurotoxin.

### 2.5. Determination of Efficacy of the Formulation

The efficacy of the formulated neurotoxin was evaluated using the Digital Abduction Score (DAS) and rota-rod assay. All experimental procedures were approved by the Institutional Animal Care and Use Committee (IACUC). Groups of five BALB/c mice were administered topical doses of neurotoxin (0 to 100 units). Mice in the negative control group received only PBS, and one cohort received unformulated neurotoxin. Mice were acclimatized for 7 days under a 12:12 h light–dark cycle [18] and anesthetized using a ketamine–xylazine mixture before neurotoxin application to the hind limbs. The DAS assay [19] was performed to assess functional recovery. In this assay, mice were suspended by the tail, allowing the hindlimbs to hang freely while the forelimbs rested on a workbench. The degree of digit abduction was scored on a five-point scale (0, normal; 4, maximal reduction in digit abduction and leg extension) by an observer blinded to the treatment. Measurements were taken every 24 h, and the average of three independent observers’ assessments was used for analysis, with standard deviations included in the results.

### 2.6. Motor Coordination Assay (Rota-Rod Assay)

To assess the effects of neurotoxin on motor function, mice were trained for 1 week on the rota-rod device (S.D. Scientific Works, Kolkata, India), with an 8 cm diameter rod. Mice were tested every other day, and the number of rotations completed before falling was recorded. The average number of rotations for each mouse was used to assess the effect of the administered neurotoxin dose.

### 2.7. Franz Cell Assay

Human skin permeation studies were conducted using vertical glass Franz diffusion cells (Syncbio Research Pvt. Ltd., Ahmedabad, India). BoNT/A was biotinylated using the EZ-Link™ sulfo-NHS-LC-Biotinylation Kit (Thermo Scientific, Waltham, MA, USA) according to the manufacturer’s protocol. Human skin (donated by Science Care, Phoenix, AZ, USA) was mounted in the diffusion cells and skin surface measured using a calibrated infra-red thermometer. Skin surface temperatures was within 32 ± 1 °C. All the experiments were performed in environmentally controlled temperature at 21 ± 2 °C and 50 ± 20% relative humidity to simulate in vivo conditions. Trans-epidermal water loss (TEWL) and skin temperature were monitored, with the skin thickness measured using a calibrated micrometer screw gauge. For each experiment, 30 µL of formulated neurotoxin (doses of 25, 50, and 100 units) was applied to the donor compartment, and receptor medium (PBS, pH 7.4) was replenished at designated time points (0, 1, 2, 3, 4, 6, 8, 12, 16, 20, 24, 28, 32, and 36 h). Biotinylated neurotoxin in the receptor compartment was quantified using ELISA with streptavidin-HRP. The data obtained were utilized to generate several graphs, including (1) cumulative transport as a function of time (hours), (2) cumulative transport per unit area over time, (3) rate of toxin transport across the skin (in units per hour per unit area) relative to time, and (4) percentage recovery of toxin relative to the amount administered. The percentage recovery of toxin was calculated by dividing the cumulative toxin recovered (measured in units via ELISA) by the total amount of toxin applied.

### 2.8. Anti-Hyperhidrosis Experiment

BALB/c mice (12–14 weeks old, weighing approximately 30 g) were used for the anti-hyperhidrosis model. Mice were acclimatized for 7 days before the experiment and anesthetized with ketamine–xylazine. Pilocarpine injection (5 mg/kg, intraperitoneally) was used to induce sweating. The neurotoxin formulations and controls were applied topically to the gastrocnemius muscle, and DAS scores were measured 48 h post-application by three independent observers. After 48 h, sweating was visualized using a starch–iodine test (Sigma Aldrich). Both paws were coated with iodine and starch suspension, and sweating was induced by pilocarpine injection. The areas of sweat spots were quantified using ImageJ (version 1.54 e) software. As a positive control, mice were administered 5 units of lyophilized BoNT/A, formulated with sucrose and human serum albumin (HSA), and resuspended in 0.9% saline.

## 3. Results and Discussions

### 3.1. Botulinum Neurotoxin (BoNT/A) Production and Characterization

**Figure 1:**
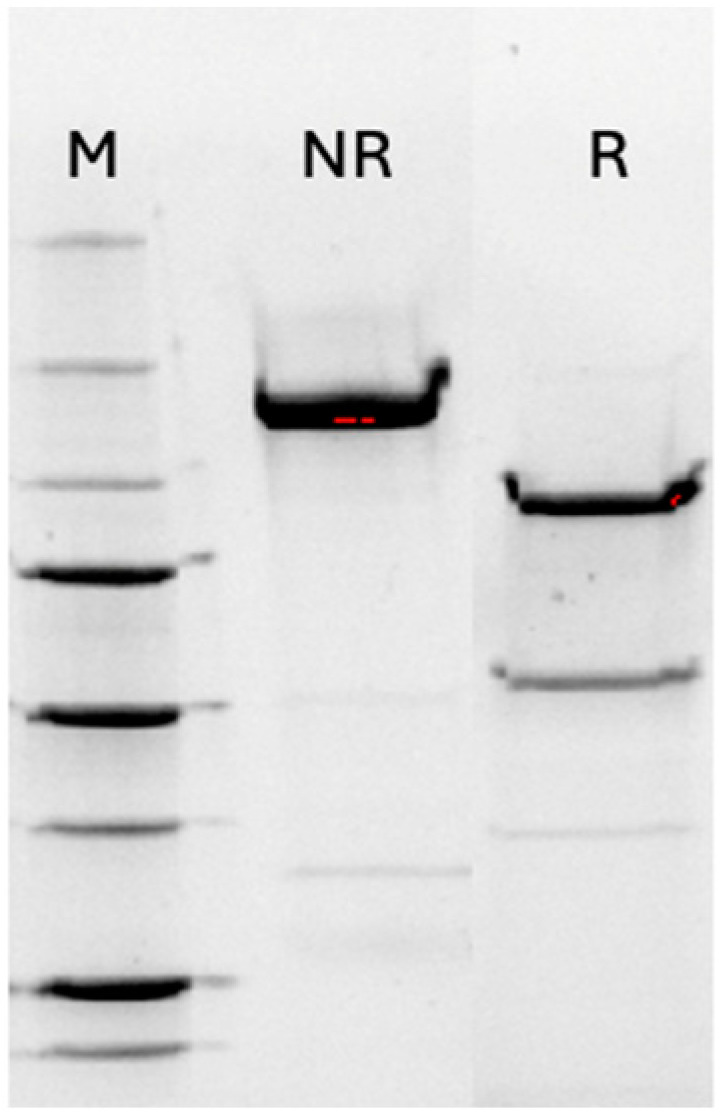
SDS-PAGE analysis of 150 kDa BoNT/A in reduced (R) and non-reduced (NR) conditions: SDS-PAGE analysis of the 150 kDa BoNT/A was performed under both reduced (R) and non-reduced (NR) conditions. The molecular weight markers (M) used in the analysis included bands at 260, 160, 100, 75, 50, 37, and 25 kDa, among others.

Botulinum neurotoxin A (BoNT/A; 150 kDa) was produced as previously described. Briefly, the complex was isolated from crude extract using a DEAE-A50 column (Figure 1). The resulting BoNT complex exhibited a typical seven-band pattern on SDS-PAGE, consisting of the neurotoxin binding protein (NBP) or non-toxic non-hemagglutinin activity (NTNHA), along with the hemagglutinin (HA) proteins and the neurotoxin itself. The complex was subsequently purified to yield the 150 kDa BoNT/A neurotoxin (Figure 1). The purified neurotoxin was confirmed to be BoNT/A using an anti-BoNT/A antibody (BB256, Prime Bio, Inc., Dartmouth, MA, USA).

To determine the LD_50_ of the neurotoxin preparation, a mouse bioassay was performed, which revealed an LD_50_ of 25 pg/kg (1 unit) for BoNT/A. Botulinum neurotoxin A is typically produced in a nicked form (dichain), and upon reduction, it separates into the heavy chain (binding domain, 100 kDa) and light chain (50 kDa). This separation was confirmed by SDS-PAGE (Figure 1).

### 3.2. Formulation Development

The formulation was prepared by first mixing sodium hyaluronate, propylene glycol, Tween-80, and saponin in water under continuous stirring to create the water-soluble phase. Once fully mixed, phenoxyethanol and tricaprylin were added while stirring continuously to form the emulsion. Several compositions were prepared and screened for their effectiveness, but data for only the optimal formulation are presented here. The formulations differed in their content of saponin and/or hydrogenated castor oil. The best formulation, referred to as S-I, contained saponin but excluded hydrogenated castor oil.

To prepare the final formulation, BoNT/A neurotoxin was dissolved into the emulsion and stirred for 15–20 min at room temperature. The activity of the neurotoxin in the formulation was assessed by incubating 30 nM of formulated neurotoxin with 2 µM of SNAMP (a chimeric substrate composed of the SN2 domain of SNAP-25 and the VAMP2domain) at 37 °C for 1 h. The neurotoxin retained full activity with respect to the SNAMP substrate.

**Figure 2:**
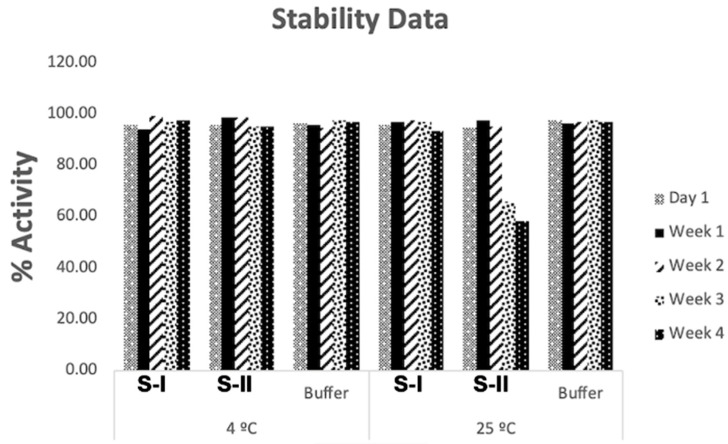
Stability data of BoNT/A in formulations (S-I) and (S-II): stability of BoNT/A in formulation S-I and formulation S-II was evaluated by storing samples at 4 °C and 25 °C. Neurotoxin activity was assessed by measuring the percentage of activity using an in-house SNAMP substrate.

To assess the stability of the neurotoxin, formulations were stored for one month at 4 °C (Figure 2). Formulation S-I (without hydrogenated castor oil) and formulation S-II (without both hydrogenated castor oil and sodium hyaluronate) showed that BoNT/A remained stable at 4 °C for the full duration. However, when stored at 25 °C, neurotoxin in S-II lost significant activity after two weeks, while in S-I, activity was maintained at least for four weeks at both temperatures. The key difference between S-I and S-II was the presence or absence of sodium hyaluronate and hydrogenated castor oil, respectively.

### 3.3. Transdermal Delivery of Neurotoxin

To assess the transdermal transport of the formulated neurotoxin, we topically administered varying concentrations of the neurotoxin to the hind legs of mice. Following application, the degree of local muscle paresis was evaluated using the Digital Abduction Score (DAS), which measures toe-spread (digit abduction). Initially, each cohort of mice received a topical dose of formulated BoNT/A (ranging from 5000 units to 10 units, with a gradual decrease) on the left gastrocnemius muscle. The right hind leg was treated with the vehicle as a control. Effectiveness was assessed by measuring the DAS score.

During initial screening, three mice were treated with unformulated neurotoxin, but no DAS score was observed. Formulated neurotoxin was effective at concentrations as low as 25 units, whereas no DAS score was observed at 10 units. Given the small cohort size (two mice per concentration) during screening, a more systematic study was performed with a larger sample size (10 mice per group) at four concentrations: 10, 25, 50, and 100 units, in addition to the vehicle control.

**Figure 3:**
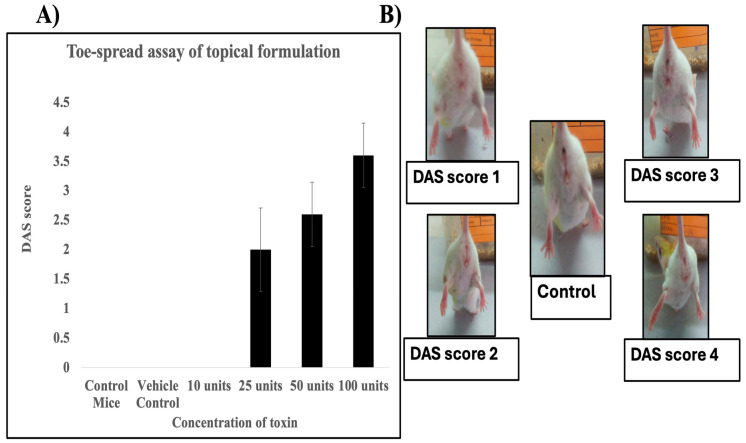
Transdermal delivery of formulated BoNT/A and DAS assessment. (**A**) Dose–response curve of formulated BoNT/A based on the toe-spread assay. The average Digital Abduction Score (DAS) (y-axis) is plotted against the different doses of BoNT/A topically administered (x-axis). (**B**) Representative images showing the observable DAS score in mice after topical administration of formulated neurotoxin (left hind leg) and vehicle control (right hind leg). The images illustrate the degree of local muscle paresis corresponding to the DAS score.

In this study, the formulated neurotoxin was administered topically to both hind legs of each mouse, while the control group received the vehicle on both hind legs. After 48 h, DAS scores were measured and averaged across each cohort. No DAS score was recorded in mice that received either the vehicle or 10 units of formulated neurotoxin (Figure 3A). However, mice treated with 25, 50, and 100 units of the formulated neurotoxin exhibited local muscle paresis, as evidenced by the measurable DAS score (Figure 3B). The average DAS scores were 2.00 ± 0.71, 2.5 ± 0.54, and 3.6 ± 0.44, respectively, indicating a dose-dependent paralysis of the neuromuscular junction, specifically in the toe-reflex (Figure 3A).

This experiment demonstrates that the formulated neurotoxin can be effectively delivered transdermally following topical application. No penetration or activity was observed with unformulated neurotoxin.

### 3.4. Correlation Between DAS Score (Local Paralysis) and Rota-Rod Recovery Time (Motor Function)

As described earlier, the topical administration of formulated neurotoxin induced muscle paresis. To assess the onset and duration of action, mice were topically administered formulated BoNT/A at doses of 100, 50, or 25 units to both hind leg gastrocnemius muscles or with vehicle control each experiment was conducted with 4 mice). This assay was also performed with unformulated neurotoxin and IgG (2 mice each), and none of the mice exhibited muscle paresis. Their Digital Abduction Score (DAS) was 0, similar to that of the PBS control and vehicle control groups. No rota-rod assay was performed on mice treated with unformulated neurotoxin.

**Figure 4:**
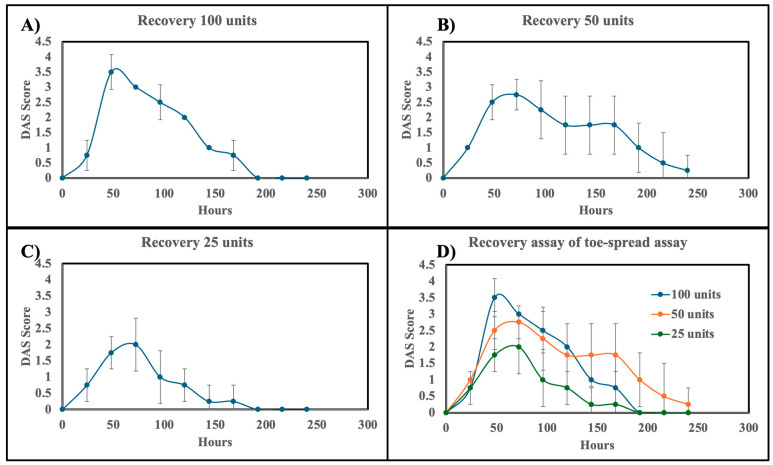
Efficacy of formulated BoNT/A as assessed by Digital Abduction Score (DAS) in mice. (**A**) Recovery assessment following topical administration of 100 units formulated neurotoxin. (**B**) Recovery assessment following topical administration of 50 units formulated neurotoxin. (**C**) Recovery assessment following topical administration of 25 units formulated neurotoxin. (**D**) Recovery assessment for all tested concentrations of formulated neurotoxin (100, 50, and 25 units).

The DAS (to assess local paralysis) and number of rotations on the rota-rod (to assess overall motor function) were recorded daily from day 0 to day 10 by three independent observers. Muscle paresis was first observed at 24 h in all mice treated with formulated neurotoxin at doses of 25 units or higher. As expected, increasing the dose resulted in a dose-dependent increase in the DAS score. The maximum DAS scores for the 100-, 50-, and 25-unit doses were reached between 48 to 72 h, with average values of 3.5, 2.5, and 2.0, respectively (Figure 4A–D). After day 4, the DAS scores began to decrease, and by days 8 to 10, all mice had DAS scores between 0.5 and 0.

**Figure 5:**
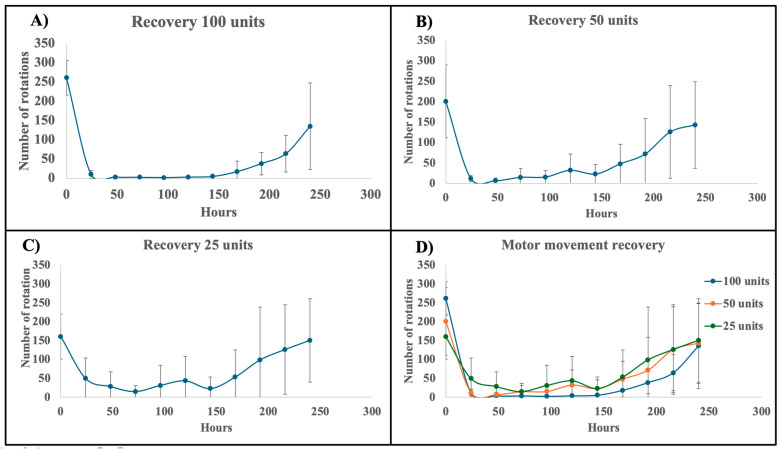
Rota-rod assay for assessment of recovery motor function recovery was assessed using the rota-rod test following topical administration of formulated BoNT/A at varying doses. (**A**) Recovery in mice administered 100 units of formulated BoNT/A. (**B**) Recovery in mice administered 50 units of formulated BoNT/A. (**C**) Recovery in mice administered 25 units of formulated BoNT/A. (**D**) Combined data from all doses (100, 50, and 25 units) illustrating the overall trend in motor recovery across groups.

The DAS score and rota-rod recovery time showed a strong correlation (Pearson correlation coefficient values are −0.5751, −0.8666, and −0.7269 for 100 units, 50 units, and 25 units, respectively) throughout the study (Figure 4 and Figure 5). As the DAS score increased, indicating muscle paresis, the number of rotations on the rota-rod decreased (Figure 4). This relationship remained consistent during the recovery phase: as motor activity recovered, the number of rotations increased, and the DAS score decreased. On average, the DAS score returned to zero around day 8, with the number of rotations beginning to increase from day 8 onwards, irrespective of the administered dose.

Notably, the rate of recovery varied with dose. Mice treated with 100 units of formulated BoNT/A showed a faster reduction in DAS score (0.44 ± 0.02 DAS per 2 days; Figure 4A) compared to those treated with 50 units (0.28 ± 0.01 DAS per 2 days; Figure 4B) and 25 units (0.22 ± 0.01 DAS per 2 days; Figure 4C). Several factors may contribute to this dose-dependent trend:

(a) Although the exact mechanism of transdermal transport is still under investigation, it is likely that the neurotoxin passes through intercellular spaces, where components of the emulsion—particularly saponin—loosen the lipid structure of the stratum corneum, facilitating the passage of the large neurotoxin molecule. For higher doses, such as 100 units, both the neurotoxin and emulsion may cumulatively disrupt the lipid bilayer, resulting in a higher and more continuous flow of the neurotoxin.

(b) Although the rate of effect is faster for higher doses, the neurotoxin effect persists longer, as indicated by the gradual recovery of motor activity, which suggests that the transdermal flow of neurotoxin continues at a sustained rate.

Interestingly, the peak DAS scores observed in our study were comparable to those induced by injectable BoNT/A1 and BoNT/A6 in gastrocnemius muscle [20].

### 3.5. In Vitro Human Skin Permeation Studies

**Figure 6:**
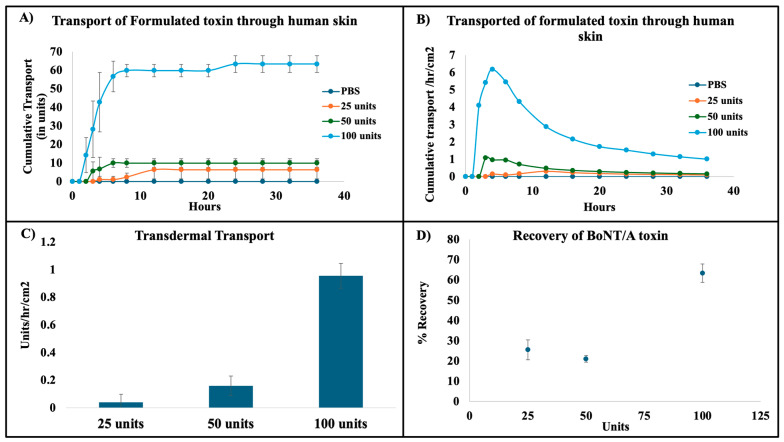
Transdermal permeation and recovery of formulated neurotoxin. (**A**) Cumulative transport of formulated neurotoxin through human skin over a 36 h period. The plot shows the cumulative amount of neurotoxin passing through the skin, with a clear dose-dependent increase in transport observed for the 100-, 50-, and 25-unit formulations. (**B**) Flux of formulated neurotoxin through human skin, calculated as the cumulative transport per unit area per hour. The maximum flux for the 100-unit formulation is observed around 6 h, after which it decreases gradually. The 50- and 25-unit formulations show similar trends, with lower peak flux values. (**C**) Transdermal transport rate per hour per unit area (units/h/cm^2^), representing the amount of neurotoxin crossing the skin barrier at each time point. The 100-unit formulation shows the highest rate of transport, followed by the 50- and 25-unit formulations, with the flux gradually decreasing over the 36 h period. (**D**) Overall recovery of neurotoxin after topical administration, showing the total recovery of neurotoxin over the 36 h study period. Higher concentrations of neurotoxin (100 units) exhibit greater recovery and distribution compared to lower concentrations (50 and 25 units), indicating a dose-dependent relationship in both permeation and recovery.

Finite dose human skin permeation studies were conducted over a 36 h period to evaluate the transdermal transport of formulated BoNT/A. The results are shown in Figure 6. The cumulative amount of neurotoxin passing through the human skin was measured over time. The data clearly indicate the passage of neurotoxin, with the highest transdermal transport observed in the formulation containing 100 units of BoNT/A, compared to the 50- and 25-unit formulations. Transport was observed to occur predominantly between 3 and 12 h after application.

The flux of neurotoxin through the skin was calculated as the cumulative transport per hour per cm^2^. For the formulation with 100 units of neurotoxin, the maximum flux was observed around 6 h, reaching approximately 6 units/h/cm^2^, after which the flux gradually decreased. The 50-unit formulation showed the highest flux (1.07 units/h/cm^2^) between 3 and 6 h, followed by a plateau that lasted until 6 h, after which the flux gradually declined. The 25-unit formulation exhibited a lower flux, which decreased after 12 h of topical application.

The overall permeation rate, calculated over the entire 36 h period, was highest in the 100-unit formulation (0.96 ± 0.09 units/h/cm^2^), followed by the 50-unit formulation (0.20 ± 0.07 units/h/cm^2^), and lowest for the 25-unit formulation (0.04 ± 0.06 units/h/cm^2^). While a clear dose-dependent increase in permeation was observed, the relationship was not strictly linear, suggesting that other factors, such as the neurotoxin itself, may influence its transport through the skin.

The recovery of neurotoxin over the 36 h period was assessed, revealing a significant dose-dependent effect. The higher flux in the 100-unit formulation correlated with better recovery and distribution. In contrast, lower concentrations of neurotoxin exhibited lower flux and recovery rates, potentially due to the limitations of the ELISA assay, which has a detection threshold of approximately 3 units. This suggests that while lower concentrations may not be detected accurately due to lower flux, the neurotoxin is still likely permeating the skin but at levels below the assay’s detection limit.

Several factors may account for the non-linearity in the dose–response relationship observed in the permeation data. These include (a) variability in skin samples (e.g., ethnicity, age, and medical history of donors), (b) the cause of death and other donor-specific factors, and (c) the quantification limitations of the ELISA-based detection system, which could fail to detect low-concentration neurotoxin accurately.

Despite these factors, the overall data suggest that the 100-unit formulation achieved the most consistent and highest transdermal permeation, with effective distribution over the 36 h period.

### 3.6. In Vivo Efficacy in Mice Model of Hyperhidrosis

To assess the efficacy of the formulated neurotoxin in treating hyperhidrosis, we utilized the foot pad area of mice, where eccrine sweat glands are concentrated, as the site for topical delivery. Hyperhidrosis was induced in the mice by intraperitoneal injection of pilocarpine (5 mg/kg body weight), a cholinergic agent that binds to muscarinic receptors on eccrine sweat glands and stimulates sweating. Sweating inhibition was evaluated using the starch–iodine test, a standard method for detecting excessive sweating associated with hyperhidrosis.

**Figure 7:**
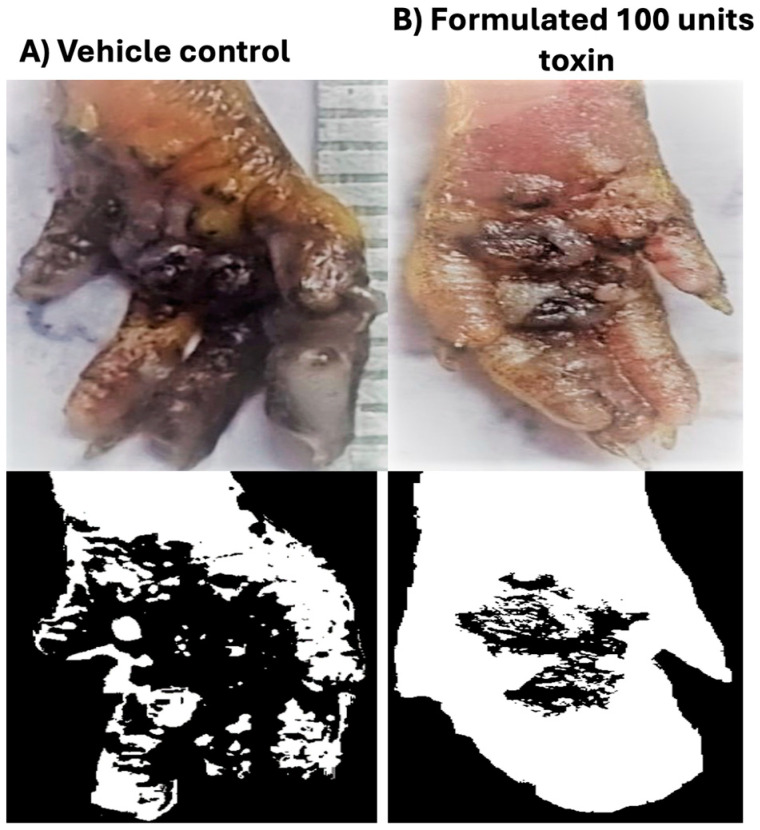
Hyperhidrosis model—effect of topically administered neurotoxin on mouse paws. (**A**) Image of the vehicle control treatment on the mouse paw (top left), showing normal sweating activity. (**B**) Image of the mouse paw treated with formulated 100 units of neurotoxin (top right), illustrating the reduction in sweating. Lower images showing paw processing and analysis using ImageJ software for quantification of sweating inhibition (lower left and lower right, respectively).

Mice were pretreated topically with either formulated BoNT/A or various controls, including vehicle, formulated human serum albumin (HSA), and neurotoxin in PBS with HSA (Table 1; Figure 7). Additionally, lyophilized neurotoxin complex, formulated with sucrose and HSA, and resuspended in 0.9% saline, was administered intramuscularly to serve as a positive control.

The topical administration of formulated BoNT/A demonstrated a significant reduction (*p* < 0.0001 with respect to control) in sweating, comparable to the intramuscular injection of neurotoxin. Specifically, the anti-hyperhidrosis effect of the 100-unit formulated neurotoxin was 88.32 ± 3.90%, which closely mirrored the 86.13 ± 4.82% reduction seen with intramuscular injection of neurotoxin. These results clearly indicate that the formulation successfully delivered the 150 kDa neurotoxin across the skin and achieved a therapeutic effect, significantly reducing excessive sweating (Figure 7).

Current treatments for hyperhidrosis involve the direct injection of botulinum neurotoxin, with dosages ranging from 50 to 200 units, depending on the area of treatment (e.g., axilla, palms, or soles of feet). Typically, the neurotoxin is reconstituted in 1.5 to 3 mL of saline and injected into multiple sites 1–2 cm^2^ apart. However, this method is associated with considerable pain, a temporary reduction in handgrip strength, and the need for repeated treatments. Surgical interventions, while providing longer-lasting results, are linked to significant risks, including postoperative complications and sexual dysfunction [21,22,23].

The present study demonstrates that the topical formulation of BoNT/A offers an effective, non-invasive alternative for the treatment of hyperhidrosis, with the potential to reduce the need for painful injections and their associated side effects.

## 4. Conclusions

In this study, we developed a novel topical formulation capable of delivering large biomolecules, such as botulinum neurotoxin A (BoNT/A, 150 kDa), across the skin. The formulation demonstrated the ability to deliver not only the large protein but also other molecules like biotinylated IgG (with a transport flux of 1.36 ± 0.35 units/h/cm^2^) through human skin. Key innovations of this formulation include the following: (a) stability: the neurotoxin remained stable in the liquid formulation for extended periods; (b) effective transdermal delivery: the formulation facilitated the transdermal delivery of proteins without the need for any external stimulation or aids, allowing for dose-dependent penetration through the skin’s thick layers; and (c) therapeutic efficacy: animal studies confirmed that the formulation effectively delivered the neurotoxin, inducing the desired therapeutic effects, including significant reductions in sweating (as observed in a mouse paw model), comparable to intramuscular injection of neurotoxin.

This formulation provides several advantages over current delivery methods, including a painless, convenient, and non-invasive system for localized therapy. It offers precise, targeted delivery with minimal discomfort for patients, presenting a promising alternative to injectable neurotoxin treatments, which are typically associated with pain and require repeated administration. Additionally, to the best of our knowledge, this is the first delivery platform capable of effectively delivering large biomolecules transdermally, making it a versatile tool for a range of therapeutic applications.

While technology shows promise, one concern is the diffusion of biomolecules through the skin. However, in the case of hyperhidrosis, where neurotoxin is administered to large skin areas, the topical delivery formulation appears to offer diffusion characteristics similar to injectable neurotoxin. Further studies are needed to ensure the safety and long-term efficacy of this technology for broader clinical use.

## Figures and Tables

**Table 1:** Hyperhidrosis experiment for formulated neurotoxin (100 units). Four controls were used: (a) vehicle control: mice treated with the vehicle (no neurotoxin) to assess baseline sweating levels; (b) formulated HSA (human serum albumin): mice treated with formulated human serum albumin (HSA) to evaluate the effect of the formulation without the neurotoxin; (c) neurotoxin in PBS with HAS: mice treated with botulinum neurotoxin in phosphate-buffered saline (PBS) with HSA, to assess the effect of neurotoxin in the formulation without active delivery components; (d) a positive control of IM administered neurotoxin: mice treated with intramuscularly (IM) administered botulinum neurotoxin as a positive control, representing the conventional method of neurotoxin delivery.

Group	Animal No.	Left Leg DAS Score	Right Leg DAS Score	Average DAS Score	Anti-Hyperhidrosis Effect (Average % Area)
Vehicle control	1	0	0	0	48.88 ± 7.55
	2	0	0	0	
	3	0	0	0	
	4	0	0	0	
	5	0	0	0	
Formulated neurotoxin (100 units)	1	2	2	2	88.32 ± 3.90
	2	2	2	2	
	3	2	2	2	
	4	3	3	3	
	5	2	1	1.5	
Formulated HSA in the vehicle	1	0	0	0	NA
	2	0	0	0	
	3	0	0	0	
	4	0	0	0	
	5	0	0	0	
100 units of neurotoxin formulated in HSA	1	0	0	0	NA
	2	0	0	0	
	3	0	0	0	
	4	0	0	0	
	5	0	0	0	
Intramuscular injection of lyophilized BoNT/A complex formulated with sucrose, HSA and resuspended in 0.9% saline (5 units)	1	1	2	1.5	86.13 ± 4.82
	2	1	1	1.0	
	3	1	1	1.0	
	4	2	1	1.5	
	5	1	2	1.5	

## Data Availability

All the data are published in the figures.
